# Supplementary material for: Research coordinators’ perspectives on recruitment of minoritized people with cystic fibrosis into clinical trials
Source: BMC Pulm Med. 2025 Jul 4;25:325. doi: 10.1186/s12890-025-03707-9 (PMC12228391; doi:10.1186/s12890-025-03707-9)
Supplement: Supplementary file 1 — Supplementary Material 1 [file 12890_2025_3707_MOESM1_ESM.docx]

**Online Supplement**

**CF Clinical Trial Recruitment Practices with Nonwhite and/or Hispanic People Survey Items**

1. Are you a research coordinator responsible for recruiting study participants in CF clinical trials? LOGIC: if yes proceed to question #2 and then to #3, if no proceed to question #2 then to #15

Yes

No

1. How long have you been a research coordinator working on CF clinical trials?
   1. Less than 2 years
   2. 2 – 5 years
   3. 6 – 10 years
   4. Greater than 10 years

The next few questions are designed to help us know more about your experience and suggestions around **building a culture of research** with diverse populations.

1. How much experience do you have in discussing CF research in general with nonwhite and/or Hispanic people?

LOGIC if a or b, go to question #4, then #5, then #7 if c or d go to question #6, then #7

- 1. None
  2. Very little
  3. A moderate amount
  4. A great deal

1. What are the reasons why you have no or little experience in discussing CF research with nonwhite and/or Hispanic people? (Check all that apply) (a through e are choices, f everyone replies to)
   1. Lack of resources or staff
   2. Lack of comfort or knowledge in how to do so
   3. Language barriers that limit ability to communicate and limited or no availability of translators
   4. Center has no nonwhite and/or Hispanic patients
   5. Please elaborate on your response – Open text box
2. Which of the following would you find helpful when discussing CF research with nonwhite and/or Hispanic people? (check all that apply) open text box optional
   1. Having a trusted team member introduce the idea of research to the patient first
   2. Using printed materials to aid in the conversation (infographics and the like)
   3. Having printed materials translated into specific languages
   4. Being able to provide transportation, beyond travel reimbursement, for study visits (examples include Uber Health or other medical transportation services)
   5. Being able to providing extended hours or weekends for study visits
   6. Having access to printed educational materials on clinical trial participation relevant to concerns of nonwhite and/or Hispanic people
   7. Having RC training in cultural competency
   8. Other (open text box)
3. Which of the following have you found helpful when discussing CF research with nonwhite and/or Hispanic people? (check all that apply) open text box optional
   1. Having a trusted team member introduce the idea of research to the patient first
   2. Using printed materials to aid in the conversation (infographics and the like)
   3. Having printed materials translated into specific languages
   4. Being able to provide transportation, beyond travel reimbursement, for study visits (examples include Uber Health or other medical transportation services)
   5. Being able to providing extended hours or weekends for study visits
   6. Having access to printed educational materials on clinical trial participation relevant to concerns of nonwhite and/or Hispanic people
   7. Having RC training in cultural competency
   8. Other (open text box)

The next few questions are designed to help us know more about your experience and suggestions around **recruiting diverse populations for a specific study**.

1. How much experience do you have in discussing specific CF clinical trials with nonwhite and/or Hispanic people?

LOGIC: if a or b go to #8, then #9 then #11, if c or d go to #10, then #11

- 1. None
  2. Very little
  3. A moderate amount
  4. A great deal

1. Have any of the following interfered with your ability to approach nonwhite and/or Hispanic people to discuss specific CF clinical trials? (mark all that apply)? (a – e are choices, everyone responds to f)
   1. Lack of resources or staff
   2. Lack of comfort or knowledge in how to do so
   3. Language barriers that limit enrollment potential
   4. Center has no nonwhite and/or Hispanic patients

e. Center has no nonwhite and/or Hispanic patients who qualified for our CF studies

f. Please elaborate on your response Open text box

1. Which of the following would you find helpful when discussing specific CF clinical trials with nonwhite and/or Hispanic people? (check all that apply) open text box optional
   1. Having a trusted team member introduce the idea of research to the patient first
   2. Using printed materials to aid in the conversation (infographics and the like)
   3. Having printed materials translated into specific languages
   4. Being able to provide transportation, beyond travel reimbursement, for study visits (examples include Uber Health and other medical transportation services)
   5. Providing extended hours or weekends for study visits
   6. Having access to printed educational materials on clinical trial participation relevant to concerns of nonwhite and/or Hispanic people
   7. Having RC training in cultural competency
   8. Other (open text box)
2. Which of the following have you found helpful when discussing a specific CF clinical trial with nonwhite and/or Hispanic people? Open text box optional
   1. Having a trusted team member introduce the idea of research to the patient first
   2. Using printed materials to aid in the conversation (infographics and the like)
   3. Having printed materials translated into specific languages
   4. Being able to provide transportation, beyond travel reimbursement, for study visits (examples include Uber Health and other medical transportation services)
   5. Providing extended hours or weekends for study visits
   6. Having access to printed educational materials on clinical trial participation relevant to concerns of nonwhite and/or Hispanic people
   7. Having RC training in cultural competency
   8. Other (open text box)

The next few questions are designed to help us know more about your experience and suggestions around **successfully enrolling study participants from diverse populations into trials**.

1. How much experience have you had in successfully enrolling nonwhite and/or Hispanic people into a CF clinical trial?

LOGIC: if a or b, then go to #12, then 14, then 15, if c or d go to #13, then #14 and then 15

- 1. None
  2. Very little
  3. A moderate amount
  4. A great deal

1. Why do you think you have not been successful in enrolling nonwhite and/or Hispanic people into CF clinical trials? Open text box
2. Why do you think you have been successful in enrolling nonwhite and/or Hispanic people into CF clinical trials? Open text box
3. What do you see are the key barriers to enrolling nonwhite and/or Hispanic people in CF clinical trials? Open text box
4. What do you think would help teams be more effective in enrolling nonwhite and/or Hispanic people in CF clinical research? Open text box
5. Key themes and barriers already identified from early listening efforts with community members of color include:

- Lack of trust between people of color with CF and the broader CF Community, as well as the CF Foundation
- Lack of representation across many areas (Care center/CFF staff, media, clinical trials)
- Lack of education in clinical settings on diagnosing and treating Black people and other people of color with CF
- Delayed Diagnosis / Misdiagnosis
- Lack of CFF support and visibility for people of color affinity groups

What are your thoughts and reactions to this (open text box)?

1. Is there anything else you would like to share with us on this topic? Open text box
2. If we have further questions regarding your experience may we contact you? If yes, please leave your name and the best way to get in touch with you. If no, please skip this question. (Open text box)
